# Oral Microbiota: The Influences and Interactions of Saliva, IgA, and Dietary Factors in Health and Disease

**DOI:** 10.3390/microorganisms11092307

**Published:** 2023-09-13

**Authors:** Daisuke Nagakubo, Yuichiro Kaibori

**Affiliations:** 1 Division of Health and Hygienic Sciences, Faculty of Pharmaceutical Sciences, Himeji Dokkyo University, 7-2-1 Kamiohno, Himeji 670-8524, Hyogo, Japan; 2 Laboratory of Analytics for Biomolecules, Faculty of Pharmaceutical Science, Setsunan University, 45-1 Nagaotoge-cho, Hirakata-shi 573-0101, Osaka, Japan; yuichiro.kaibori@setsunan.ac.jp

**Keywords:** oral microbiota, saliva, IgA, fermented food

## Abstract

Recent advances in metagenomic analyses have made it easier to analyze microbiota. The microbiota, a symbiotic community of microorganisms including bacteria, archaea, fungi, and viruses within a specific environment in tissues such as the digestive tract and skin, has a complex relationship with the host. Recent studies have revealed that microbiota composition and balance particularly affect the health of the host and the onset of disease. Influences such as diet, food preferences, and sanitation play crucial roles in microbiota composition. The oral cavity is where the digestive tract directly communicates with the outside. Stable temperature and humidity provide optimal growth environments for many bacteria. However, the oral cavity is a unique environment that is susceptible to pH changes, salinity, food nutrients, and external pathogens. Recent studies have emphasized the importance of the oral microbiota, as changes in bacterial composition and balance could contribute to the development of systemic diseases. This review focuses on saliva, IgA, and fermented foods because they play critical roles in maintaining the oral bacterial environment by regulating its composition and balance. More attention should be paid to the oral microbiota and its regulatory factors in oral and systemic health.

## 1. Introduction: The Complex Relationship between the Microbiota and Health

The microbiota is a cluster of microorganisms, including bacteria, archaea, fungi, and viruses, that live symbiotically with hosts, such as humans and mice, within a specific environment [1]. The microbiota in the digestive tract, such as the small intestine and colon, are well-known as representative examples. Metagenomic analyses based on the recent development in next-generation sequencing technology have revealed that the microbiota adapts to various environments and forms unique microbial communities in host tissues, such as those of humans [2] and other animals, including some new examples of wild gibbons [3], calves [4], and seagulls [5]. Interestingly, humans co-evolve with the microbiota [6,7,8]. This co-evolution is an interdependence in which the microbiota provides many of the biological functions that humans need to survive, while humans provide a habitat for the microbiota. The microbiota performs crucial functions in the human body, such as digestion and absorption of nutrients, regulation of the immune system, protection of the intestinal wall, and production of neurotransmitters [9]. On the one hand, the human body (particularly the intestine) provides a stable habitat and nutrients for the microbiota. Thus, humans and their microbiota depend on each other to live together and adapt, survive, and thrive through each other’s presence. This relationship involves traits that individuals retain throughout their lives and are passed on from generation to generation. Genetic characteristics are maintained to some extent through the transmission of the microbiota from parent to offspring, and they can adjust and change through interactions with the environment [10].

As a matter of public health, many studies have shown that the balance of bacteria that make up the microbiota is closely related to the host’s immune system [11]. Interactions between the immune system and the microbiota affect human health and disease. Changes in the gut microbiota are thought to be involved in inflammatory bowel diseases (IBDs), especially Crohn’s disease (CD) and ulcerative colitis (UC). A healthy gut microbiota interacts with the host immune system to maintain homeostasis. However, in patients with IBD, changes in the composition of the gut microbiota (dysbiosis) induce an inflammatory response and contribute to disease persistence and progression. For instance, Ni et al. demonstrated that the gut microbiota of patients with CD differs from that of healthy individuals. Particularly, the gut microbiota of patients is less diverse and contains an excess of specific pathogenic bacteria [12]. The immune system and the gut microbiota interact closely and play a critical role in immunoregulation. For example, microbiota alterations may play a role in immune-related diseases such as rheumatoid arthritis, type 1 diabetes, multiple sclerosis (MS), and systemic lupus erythematosus (SLE). Belkaid and Hand discussed the influence of the gut microbiota on the host’s immune system and inflammation in detail. They pointed out that the microbiota affects the development and function of T cells, which could potentially lead to the onset of immune diseases [13]. The gut microbiota also affects the development of allergies. The gut microbiota of infants changes rapidly during the first year of life, and its composition during this critical period can influence the risk of allergy development. Stokholm et al. demonstrated that a high diversity of gut bacteria during infancy is associated with a reduced risk of developing asthma later in life [14]. In addition, there was a recent review of the respiratory microbiota and asthma in immune diseases [15]. Through these studies, it is clear that the composition of the microbiota is deeply involved in the health of the host and the onset of diseases. Therefore, understanding and manipulating the microbiota could open new avenues for preventing and treating various immune-related diseases. Recently, researchers have applied the results of microbiota analyses to human treatments. The application of the microbiota in treatment is a recent attempt to improve human health by leveraging its potential capabilities. This application enhances or modulates the gut microbiota. Probiotics, for one application, are living microorganisms that confer health benefits. Species of *Lactobacillus* and *Bifidobacterium* are the most commonly used probiotics. These microorganisms are used to improve conditions such as irritable bowel syndrome and gut microbiota imbalances. However, the results vary among individuals owing to the different responses per person and interactions among microbes [16]. Fecal transplantation is a technique for rebuilding a patient’s gut microbiota using feces from a healthy donor. This method is primarily used in cases of *Clostridium difficile* infections and IBD, where conventional treatments are ineffective. Its efficacy and safety were demonstrated in the initial clinical trials of fecal transplants [17,18,19]. In addition, prebiotics [20] and postbiotics [21] could be helpful in terms of the gut microbiota and host health promotion. Prebiotics are non-digestible food ingredients that facilitate the growth and activity of beneficial bacteria in the gut. Some dietary fibers and oligosaccharides, such as galactooligosaccharides and fructooligosaccharides, are the most common prebiotics. They promote the growth of certain healthy gut bacteria and improve health. Postbiotics are bioactive microbiota-derived components, such as metabolic products and cell wall components of microbes. They suppress the growth of pathogenic bacteria and enhance the intestinal barrier function. Because of this evidence, analyses of the relationship between the microbiota and disease onset will further accelerate.

Thus, the accumulation of these studies will lead to further advances in disease prevention and treatment strategies. However, when considering the effects of the microbiota on host health, recent studies, especially those on the intestinal tract [22,23], have shown that the composition of the microbiota changes intensely depending on various factors, such as dietary habits, food preferences, and the sanitary conditions of the living environment. For example, David et al. [24] demonstrated that plant- and animal-based diets cause significant shifts in gut microbiota composition. Animal-based diets increased *Bilophila*, which is a bile-tolerant microorganism. These changes were observed just days after the dietary changes were made. Thus, the gut microbiota can change rapidly depending on the type of diet. De Filippis et al. [25] compared the gut microbiota of omnivores and vegetarians and showed that each group had a dominant microbial population. Furthermore, in addition to diet, hygiene status also affects the gut microbiota. Antibiotic use can temporarily or permanently alter the gut microbiota [26]. Overuse of antibiotics could potentially eradicate specific bacterial populations and lead to an increase in the number of resistant bacteria. Therefore, a molecular understanding of these factors is crucial for artificially controlling the composition of the microbiota for therapeutic and preventive purposes.

The digestive tracts of mammals are typically inhabited by bacterial flora. The oral cavity opens directly to the outside as an entrance, and the properties of the microbiota tend to be different from those of the intestinal tract. In addition, temporary changes in pH and salt concentration in the oral cavity due to food nutrients and alcohol intake, as well as exposure to external pathogens, have the potential to frequently change the oral environment. The oral cavity contains such situations and forms a specific environment compared to other digestive tracts, such as the gut, which are relatively stable. Periodontal disease and dental caries are well-known pathological oral conditions that exist as problems unique to the oral cavity. Recent studies have revealed a relationship between these oral environments and the oral microbiota. We describe them later. As described in the next section, the microbiota fluctuates due to various factors, such as nutrients and specific diseases, and strongly affects the oral environment [27,28]. Recent studies have revealed that the oral microbiota is involved in the development of diseases in other organs of the body [29]. This review focuses primarily on bacteria within the oral microbiota in the oral cavity. We have also organized and outlined the factors that cause environmental changes and their roles in host defense.

## 2. Overview of Factors Affecting the Oral Microbiota

### 2.1. Functional Importance of the Intestinal and Oral Microbiota and Their Impact on Health

The mucous membranes of the digestive tract are in contact with the external environment. The epithelium of the digestive tract, which forms the border, occupies the largest surface area of the body and is constantly exposed to various stimuli. Thus, various external stimuli that may alter the growth and adaptation of individual bacteria could directly and profoundly affect the microbiota. Numerous studies have reported that various bacteria live in the oral cavity and gastrointestinal tract to form the microbiota. For example, 100 trillion bacteria inhabit the gut [30]. The commensal bacteria that constitute the microbiota of representative human sites and their associated characteristics are as follows. The human colon has the richest microbiota, primarily composed of bacteria from the *Bacteroidetes* and *Firmicutes* phyla. These bacteria help extract energy from food, regulate the immune system, and maintain gut health [31,32]. The small intestine’s microbiota has fewer types and numbers than the colon, including *Lactobacillus* [31,33]. The oral microbiota is highly diverse, with around 700 types of bacteria, and the main species include *Streptococcus*, *Neisseria*, *Veillonella*, *Prevotella*, and *Haemophilus* [34,35,36,37]. The respiratory microbiota primarily consists of bacteria such as *Prevotella*, *Veillonella*, and *Streptococcus* [34,38,39]. The skin microbiota varies depending on the skin site, but primarily includes *Cutibacterium*, *Staphylococcus*, and *Corynebacterium* [40,41,42]. An imbalance in the microbiota (also called dysbiosis) can contribute to the development of chronic diseases. As for dysbiosis, its relevance to the disease is emerging in the nervous system, as evidenced by decreased gut microbiota diversity in a rat model of depression [43].

Additionally, gut microbes are closely related to host health, especially from the viewpoint of nutrition, which supports host activity. One example is the supply of “vitamins” to hosts. Vitamins are essential nutrients that humans cannot produce by themselves, or even if they can, in small amounts. Therefore, humans must obtain vitamins from external sources, such as dietary nutrients. Gut microbes that reside in the colon are also sources of vitamins and play a vital role in their production and metabolism. In particular, the synthesis of B-group vitamins (such as thiamine, riboflavin, niacin, pantothenate, pyridoxine, biotin, folate, and cobalamin) and menaquinone (vitamin K2) by the gut microbiota is essential for humans [44,45]. Regarding B-group vitamins, the production of each vitamin varies at the species and strain levels, whereas menaquinone is produced by bacteria such as *Bacteroides* species [46]. Thus, humans establish a symbiotic relationship with the gut microbes. While these microbes obtain the nutrients they need for survival from the food humans consume, humans, in turn, benefit from these gut microbes. As shown in this example, although bacteria can benefit human health, researchers have found that differences in the balance and diversity of individual bacteria constituting the microbiota influence diseases. For example, researchers have reported changes in the gut microbiota composition of patients with IBD, broadly divided into two types, CD and UC, compared to healthy individuals [47].

In addition to diseases related to the gut, researchers have found that changes in the composition of the gut microbiota are related to systemic diseases. An example is the connection between gut microbes and obesity. Studies have demonstrated that transplanting bacteria from the feces of obese mice into normal mice induces obesity (an increase in body fat) [48]. A cohort study in Ghana involving HIV-infected individuals and sex- and age-matched HIV-uninfected counterparts revealed that the diversity in the gut microbiota in HIV-infected individuals was significantly lower than that in uninfected individuals [49]. From the perspectives gained from these examples, constructing a microbiota composition in which beneficial bacteria predominate and maintaining the balance of gut microbes is crucial for promoting the host’s health. The microbiota shaping the oral environment can also affect the gut environment. One study indicated that oral commensal bacteria could colonize the gut and potentially contribute to the onset of IBD [50]. From another perspective on the oral microbiota and health, a recent report investigating the influence of the maternal oral microbiota on the formation of the oral microbiota in healthy infants suggested that infants may be colonized with an oral microbiota different from that of their mothers at birth. Further research is needed to determine how establishing an early oral microbiota influences health over time [51].

### 2.2. The Role of Saliva in Regulating the Oral Microbiota

The oral cavity provides an optimal growth environment for many bacteria because of the stable maintenance of an appropriate temperature and humidity [52]. Therefore, a diverse range of bacteria is present in the oral cavity. Consequently, the human oral environment forms a complex system that affects health status and oral hygiene. The relationship between periodontal disease or dental caries and the oral microbiota has been investigated considering these issues. Recent research has confirmed that the oral microbiota plays a considerable role in the onset of periodontal disease and dental caries. Periodontal disease, including gingivitis and periodontitis, is a chronic inflammatory disease caused by dysbiosis of the oral microbiota. From the viewpoint of oral hygiene and immune response, it has been reported that IL-6, an inflammatory cytokine in the saliva, could be a marker for estimating inflammation in the gingiva and oral cavity [53]. In a healthy state, the oral microbiota consists of a variety of bacterial species that coexist in balance. However, when this balance is disrupted, harmful bacteria, such as *Porphyromonas gingivalis*, *Tannerella forsythia*, and *Treponema denticola*, can proliferate, leading to the onset of periodontal disease. These bacteria invade the gum tissue, causing inflammation, and potentially leading to tooth loss [54]. Dental caries (tooth decay) is also related to the oral microbiota. One of the key culprits of dental caries is *Streptococcus mutans* (*S. mutans*). This bacterium ferments dietary sugars to produce lactic acid, which can demineralize tooth enamel and lead to caries [55]. A review by Gomez and Nelson illustrated the shaping process of the early oral microbiota, the complex nature of caries-related microbial communities, including *S. mutans*, and the environmental factors contributing to microbial imbalance (dysbiosis) that could lead to the disease [55]. From a dental perspective, D’Ambrosio et al. pointed out that removable and fixed dental prostheses on teeth and implants can make the oral environment more complex by predisposing patients to bacterial plaque formation on their surfaces [56].

Saliva is a component of the oral environment and is involved in the development of periodontal disease and dental caries. If the amount and components of saliva are not appropriate, the balance of the oral microbiota will be disrupted, and the risk of developing oral diseases, such as dental caries and periodontal disease, will increase. For instance, when saliva secretion decreases, the cleansing function of the oral cavity is reduced, and food residues become more susceptible to decomposition by bacteria. Consequently, acidic substances are produced, which increase the risk of caries. In addition, an imbalance in salivary composition can lead to a relative increase in the number of pathogenic bacteria associated with periodontal disease.

Saliva, secreted from the salivary glands, provides the “moisture” essential for bacterial growth. However, saliva secreted into the oral cavity includes antimicrobial components: agglutinins, which bind to oral bacteria such as *S. mutans* and promote phagocytosis by causing bacterial aggregation [57]; lysozymes, which cause cell lysis by breaking down the cell walls [58]; lactoferrin, which suppresses biofilm formation by stimulating twitching, a specialized form of surface motility, by iron chelation [59]; and peroxidase, which exhibits antimicrobial activity by inhibiting the bacteria’s glycolytic system [58]. In addition, saliva contains secretory IgA (sIgA), which impedes bacterial binding to the mucosa [58], and the secreted mucins MUC5B and MUC7, which aggregate bacteria and prevent their attachment to the oral mucosa [58]. Saliva containing these components maintains homeostasis between the oral mucosa and a vast number of oral bacteria, including *S. mutans*. Stress, aging [60], Sjögren’s syndrome [61], and the destructive side effects of radiation therapy for head and neck cancer [62] can disrupt the functionality of the salivary glands. Salivary gland hypofunction reduces saliva secretion into the oral cavity, resulting in considerable changes in the oral microbiota [63]. Changes in the oral microbiota can result not only in deterioration of oral health, such as dental caries, periodontal disease [64,65], and inflammation of the oral mucosa, but also in association with various systemic diseases, as reported in recent years. There are reports on disease states involving oral bacteria, such as diabetes [66], the role of *Fusobacterium nucleatum* in causing poor prognosis in colorectal cancer patients [67], the identification of *Fusobacterium nucleatum* strains in colorectal cancer tissue originating from the oral cavity [68], the uncovering of periodontal disease as a risk factor for cardiovascular diseases [69], the decrease in *Prevotella histicola* and *Prevotella oulorum* in the oral cavity of patients with rheumatoid arthritis [70], and cognitive decline [71]. Although no direct causative bacteria have been identified, researchers have implicated the involvement of the oral microbiota in oral squamous cell carcinoma [72]. Oral bacteria have been detected in various tissues throughout the body, which suggests a relationship between oral bacteria and disease onset. However, data concerning the changes in the oral microbiota associated with these diseases remain insufficient. In the future, accumulating data will create a database of changes in the oral microbiota of patients with various diseases and healthy individuals. Oral microbiota analysis can lead to the non-invasive prevention and early detection of various diseases [73].

### 2.3. The Role of IgA in Regulating the Oral Microbiota

In infectious diseases, the mouth is the primary entry route for pathogens. Therefore, besides antimicrobial substances supplied by saliva, the oral cavity contains various substances that orchestrate biological defense, such as β-defensin [74], mucous components like mucins (especially MUC1) [75], their sugar chains [76], and sIgA [77]. These substances protect the body against pathogens. sIgA, recognized as the predominant immunoglobulin in the mucosal immune system, is extensively present in the mucosa of the digestive, respiratory, and vaginal tracts and in secretory fluids such as tear fluid, saliva, and breast milk [78]. sIgA arises from the maturation of IgA produced by antibody-secreting cells (ASCs) differentiated from B cells. The intestinal tract is one of the secretion sites for large amounts of sIgA by the ASCs predominantly found in its lamina propria [79]. sIgA is a dimer formed by the joining of two IgA molecules via a polypeptide called the joining chain (J chain) produced by ASCs. In the intestine, dimeric IgA binds to a secretory component derived from the polymeric IgR, which is expressed on the basement membrane of intestinal epithelial cells. It is then taken up by epithelial cells and secreted into the apical side.

In humans, there are two IgA genes: IgA1 and IgA2. The sIgA predominantly secreted into the intestinal mucosa is IgA2. In the oral cavity, the oral mucosa and salivary glands supply sIgA through a mechanism similar to that in the intestinal tract [80,81]. As for the component of IgA in human saliva, IgA1 is predominant [79]. The chemokine CCL28 plays a substantial role in regulating the chemotaxis of IgA-ASCs in the colon. A study on CCL28-deficient mice showed an abnormal distribution and reduction in IgA-ASCs in the lamina propria of the colon and decreased IgA production capacity, resulting in aggravated dextran sulfate sodium (DSS)-induced colitis and an increased frequency of bacterial infiltration into the lamina propria of the colon compared to wild-type mice [82]. These results indirectly indicated that IgA is an essential bioprotective factor in the mucous membrane.

Notably, sIgA produced by the oral mucosa and salivary glands binds to viruses and resident bacteria that invade the oral cavity, thereby blocking their adherence to the oral mucosa [83]. Recent reports indicate that sIgA can bind not only to viruses and bacteria that have been previously encountered but also to those that have not yet caused infection [84]. Consequently, oral sIgA might play an essential role in defending against infections caused by novel pathogens such as the severe acute respiratory syndrome coronavirus 2 (SARS-CoV2). Furthermore, in addition to the previously mentioned function of sIgA in aggregating bacteria and preventing their binding to the oral mucosa, a new concept from several reports has indicated that sIgA can form a part of the mucus layer covering the oral mucosa by binding to mucin in the saliva secreted into the oral cavity and the transmembrane mucin MUC1 expressed on the surface of oral mucosal epithelial cells. This suggests that sIgA binds to oral bacteria, such as *Streptococcus mitis*, *Streptococcus oralis*, and *S. mutans*, which have epitopes recognized by sIgA, selectively recruiting these specific bacteria to the mucus layer on the mucosal surfaces of the oral cavity. Thus, it is also possible that sIgA functions as a reservoir for oral bacteria and influences the formation of the oral microbiota [85].

### 2.4. The Role of Fermented Foods in Regulating the Oral Microbiota

Meals serve as a source of nutrition for oral bacteria, and the nutrients consumed can influence the selection of bacteria that can survive in the oral cavity [86]. In contrast, research has suggested that oral bacteria may affect the dietary preferences of their human hosts by adjusting the thresholds for sweet, sour, salty, and bitter tastes [87]. Concerning this point, there has already been a review paper on the possibility that the intestinal microbiota influences human eating behavior [88]. The authors noted that microbes can cause discomfort, which alters eating behavior. Experts widely acknowledge that specific nutrients and gut bacteria play critical roles in maintaining human health [89,90] and precipitating the onset of intestinal diseases [91]. Well-recognized examples for a long time are that fermented foods and *Lactobacilli* can increase the proportion of beneficial bacteria, thus improving the gut microbiota [92]. In addition, bacterial groups such as *Lactobacillus* and *Bifidobacterium*, which act as beneficial bacteria in the gut, contribute to the prevention of diseases such as hypercholesterolemia [93], IBD [94], colon cancer [95], cardiovascular disease [96], and liver disorders [97]. Moreover, *Lactobacilli* also influence the oral microbiota in addition to the gut microbiota. Reports have indicated that *Lactobacilli* inhibit the proliferation of *S. mutans*, a bacterium that causes dental caries [98]. Furthermore, our recent analyses have revealed that Yomo gyutto, fermented Japanese mugwort (*Artemisia princeps*), a food ingredient, affected saliva production and altered the composition of the oral microbiota of mice [99]. This ingredient may also affect the microbiota in the gut in some way, extending its influence beyond the oral environment. Therefore, it is conceivable that *Lactobacilli* and various fermented foods contribute to disease prevention and health promotion by altering the oral microbiota. Many studies, including ours, have indeed reported that fermented foods modulate the microbiota of various tissues throughout the body, as in the examples mentioned above. Thus, we focused on the oral cavity and intestinal tract, which are the actively studied mammalian tissues in research on the microbiota, and summarized helpful information based on recent state-of-the-art research (Table 1).

Kombucha, yogurt, kefir, buttermilk, kvass, kimchi, sauerkraut [100], and Pyrus ussuriensis Maxim [101] increased the diversity of the gut microbiota, leading to the reduction in inflammatory biomarkers. Fermented milk, fermented Laminaria japonica, kimchi, fermented Angelica sinensis, and fermented defatted soybean each improved or prevented the following conditions via alteration of the gut microbiota in human and disease rodent models: insomnia [102], autism spectrum [103], constipation [104], hyperlipidemia [105], obesity-induced neuroinflammation [106], aging [107], memory impairment [108], and cognitive impairment and colitis [109]. Fermented Japanese mugwort (Yomo gyutto) is as mentioned above [99]. Probiotic fermented milk (Batavito) [110], fermented milk [111], probiotic petit-suisse cheese [112], probiotic yogurt [113,114,115], and Italian Grana Padano (GP) cheese [116] regulated the oral microbiota or reduced the number of oral microorganisms, resulting in the prevention or improvement of periodontal disease and dental caries. Furthermore, fermented milk altered both the oral and gut microbiota and suppressed inflammation [117].

## 3. Future Prospects: The Oral Microbiota Will Hold Promise as a Potential Biomarker for Cancers

The recent spread of coronavirus disease 2019 (COVID-19), an infectious disease caused by SARS-CoV-2, has increased public interest in research on “gut bacteria” and “immunological capacity” regarding the ability to defend against infection. Therefore, fermented foods with high value-added functional components are anticipated to become unique entities that attract increasing attention. In addition, it has become clear that the composition and diversity of the microbiota tend to decrease with aging [118]. Therefore, fermented foods and other foods that alter the variation in the oral microbiota might help prevent age-related changes. Further research on these issues regarding aging will contribute to the solution. Furthermore, in recent years, there have been many reports on the relationship between oral bacteria and various forms of cancer, such as pancreatic cancer associated with periodontitis caused by oral anaerobic bacteria [119], colorectal cancer associated with *Porphyromonas gingivalis* (regarding cancer initiation, progression, and prognosis) [120], and so on. From such perspectives, oral bacteria can be considered risk factors for diseases, including cancer, or as biomarkers and prognostic factors. Cancer is one of the most prevalent diseases in the world. Therefore, in this review, we focus on cancer and extract the findings of characteristic microbes from analyses of the microbiota in the oral cavity and tongue coatings of patients with cancer based on relatively recent publications. Table 2 summarizes the oral microbiota applications as reference examples that are candidates for the diagnosis and prevention of cancer.

In the oral cavity, where oral cancers such as oral squamous cell carcinoma were present, varieties of bacterial groups [121,122,123,124,125,126,127,128,129], specific bacteria [36,130], and fungi [131] increased within the oral microbiota. Therefore, they can serve as potential biomarkers. *Firmicutes*, *Prevotella* [132], and *Fusobacterium periodonticum* [133] increased in the oral cavity of patients with pancreatic cancer. In contrast, *Streptococcus salivarius, Streptococcus thermophilus, Streptococcus australis* [132] and *Neisseria mucosa* [133] decreased. In addition to these oral microbiota changes, regarding the risk of pancreatic cancer, while the presence of *Porphyromonas gingivalis* and *Aggregatibacter actinomycetemcomitans* increased the risk, the presence of phylum *Fusobacteria* and its genus *Leptotrichia* decreased the risk [134]. In the oral cavity of patients with colorectal cancer, *Desulfovibrio desulfuricans* [135], *Eubacterium*, *Bifidobacterium*, and *Fusobacterium* [136], *Fusobacterium, Treponema*, and *Porphyromonas* [137] increased. Besides, in the oral cavity of patients with esophageal squamous cell carcinoma, *Porphyromonas gingivalis* [138], *Bosea*, *Solobacterium*, *Gemella*, and *Peptostreptococcus* [139], *Firmicutes*, *Negativicutes*, *Selenomonadales*, *Prevotellaceae*, *Prevotella*, and *Veillonellaceae* [140] increased. In contrast, *Proteobacteria*, *Betaproteobacteria*, *Neisseriales*, *Neisseriaceae*, and *Neisseria* decreased [140]. Furthermore, *Campylobacter concisus* [141] and *Streptococcus* [142] increased the risk of gastric cancer. However, *Neisseria*, *Prevotella*, *Prevotella7*, and *Porphyromonas* decreased risk [142]. *Streptococcus* indeed increased in the oral cavity of patients with gastric cancer, in addition to *Gemella*, *Escherichia-Shigella*, and *Fusobacterium* [143]. Similarly, *Neisseria* and others (*Haemophilus*, *Faecalibacterium*, and *Romboutsia*) decreased [143]. Regarding the oral cavity of patients with other cancers, *Streptococcus* in hepatocellular carcinoma [144], *Clostridia* in breast cancer [145], *Fusobacterium periodonticum* in colorectal cancer [145], *Prevotella*, *Stomatobaculum*, *Bifidobacterium*, *Peptostreptococcaceae*, *Shuttleworthia*, and *Finegoldia* in lymph node metastasis [146] increased. However, the presence of *Corynebacterium* and *Kingella* in the oral microbiota decreased the risk of head and neck squamous cell cancer [147].

From an overview of these reports, the genus *Fusobacterium* and the genus *Prevotella* increased in many cases. According to the Human Oral Microbiome Database (HOMD) (https://www.homd.org/ (accessed on 24 August 2023)) [148,149,150], the percentage abundance of the genus *Fusobacterium* is abundant in the subgingival plaque and palatine tonsils. Besides, the percentage abundance of the genus *Prevotella* is abundant in the saliva, throat, palatine tonsils, and tongue dorsum. Meanwhile, in terms of percentage abundance in the oral cavity, including saliva, both genera of bacteria are substantially present at each site of the oral cavity. However, as shown in Table 2, multiple reports have confirmed that increased levels of these bacterial genera within the oral cavity are associated with certain cancers. Accordingly, further clarification of the detailed mechanisms, such as the relationship between changes in the number and composition ratio of bacterial species contained in each genus and the degree of cancer malignancy, is necessary. In contrast, the genus *Neisseria* was generally decreased in the oral cavity of patients with cancer. Therefore, these are characteristic findings of changes in the oral microbiota and might be candidates for diagnosing various cancers. In conclusion, it is desirable to advance our understanding of the maintenance and promotion of health, prevention of systemic diseases, and therapeutic targets associated with the oral microbiota. Moreover, it is necessary to increase awareness of the importance of the oral environment, including the oral microbiota, and to improve oral care.

## Figures and Tables

**Table 1:** The beneficial roles of fermented foods.

Tissue	Fermented Food	Influences	PMID	Reference No.
Gut	Kombucha, Yogurt, Kefir, Buttermilk, Kvass, Kimchi, and Sauerkraut	Diversification of the gut microbiota, reduction of the inflammation marker	34256014	[100]
Gut	Pyrus ussuriensis Maxim	Reduction in body weight and obesity-related biomarkers in obese rat model, diversification of the gut microbiota	36058150	[101]
Gut	Fermented milk	Regulation of the gut microbiota, prolongation of sleep time	31927503	[102]
Gut	Fermented milk	Alteration of the gut microbiota, improvement of autism spectrum in the male model mice of the autism spectrum disorder	36505260	[103]
Gut	Fermented milk	Improvement of the gut microbiota, alleviating constipation in mice	37194317	[104]
Gut	Fermented Laminaria japonica	Regulation of the gut microbiota, prevention of hyperlipidemia	34231612	[105]
Gut	Kimchi	Alteration of the gut microbiota, improvement in obesity-induced neuroinflammation in obese mice model	35840231	[106]
Gut	Fermented Angelica sinensis	Regulation of the gut microbiota, prevention of liver aging via alleviating of oxidative stress in aging mice model	36477974	[107]
Gut	Fermented defatted soybean	Regulation of the gut microbiota, improvement of memory impairment in Alzheimer’s disease mice model	30152045	[108]
Gut	Fermented soybean	Alteration of the gut microbiota, enhanced effects of donepezil against cognitive impairment and colitis in mice	34579150	[109]
Oral cavity	Fermented Japanese mugwort (Yomo gyutto)	Diversification of the oral microbiota, increment in saliva	36398739	[99]
Oral cavity	Probiotic fermented milk (Batavito)	Reduction in the total number of oral microorganisms, reduction in mineral loss in bovine enamel	25627884	[110]
Oral cavity	Fermented milk	Reduction in the total number of microorganisms, especially *Streptococcus mutans*, in saliva	28803012	[111]
Oral cavity	Probiotic *petit-suisse* cheese	Reduction in *Agreggatibacter actinomycetemcomitans* and *Porphyromonas gingivalis* in saliva	30716917	[112]
Oral cavity	Probiotic yogurt	Regulation in the oral microbiota	35066918	[113]
Oral cavity	Probiotic yogurt	Alteration of the oral microbiota	33783590	[114]
Oral cavity	Probiotic yogurt	Reduction of *Streptococcus mutans* in saliva	31821997	[115]
Oral cavity	Italian Grana Padano (GP) cheese	Reduction in the overall amount of acidophilic bacteria, reduction of the *Streptococcus mutans*/*Streptococcus sanguinis* ratio	35810305	[116]
Oral cavity and gut	Fermented milk	Alteration in the oral and gut microbiota, improvement of periodontitis as well as gut inflammation	33685682	[117]

**Table 2:** The candidates of biomarkers in cancers.

Kind of cancer	Microorganism	Feature	PMID	Reference No.
Oral cancer	*Porphyromonas gingivalis* and *Fusobacterium nucleatum*	Increase in oral cancer	31370775	[36]
Oral cancer	*Pseudomonas*, *Capnocytophaga*, and *Mycoplasma*	Increase within the oral cavity where oral cancer is present	36678424	[121]
Oral cancer and oropharyngeal cancers	*Rothia*, *Haemophilus*, *Corynebacterium*, *Paludibacter*, *Porphyromonas*, *Oribacterium*, and *Capnocytophaga*	Increase within the oral cavity where oral and oropharyngeal cancers are present	30123780	[122]
Oral squamous cell carcinoma	*Prevotella*, *Capnocytophaga*, and *Fusobacterium*	Increase within the oral cavity where oral squamous cell carcinoma is present	35433507	[123]
Oral squamous cell carcinoma	*Firmicutes*, *Fusobacteria*, *Fusobacteriales*, *Fusobacteriaceae*, and *Fusobacterium*	Increase within the oral cavity where oral squamous cell carcinoma is present	36699672	[124]
Oral squamous cell carcinoma	*Bacillus*, *Enterococcus*, *Parvimonas*, *Peptostreptococcus*, and *Slackia*	Increase within the oral cavity where oral squamous cell carcinoma is present	29184122	[125]
Oral squamous cell carcinoma	*Fusobacterium*, *Treponema*, *Streptococcus*, *Peptostreptococcus*, *Carnobacterium*, *Tannerella*, *Parvimonas*, and *Filifactor*	Increase within the oral cavity where oral squamous cell carcinoma is present	32753953	[126]
Oral squamous cell carcinoma	*Prevotella*	Increase within the oral cavity where oral squamous cell carcinoma is present	33155101	[127]
Oral squamous cell carcinoma	*Capnocytophaga*, *Haemophilus*, and *Neisseria*	Increase within the oral cavity where oral squamous cell carcinoma is present	34712209	[128]
Oral squamous cell carcinoma	*Actinobacteria*, *Fusobacterium*, *Moraxella*, *Bacillus*, and *Veillonella*	Increase within the oral cavity where oral squamous cell carcinoma is present	34485181	[129]
Oral squamous cell carcinoma	*Prevotella melaninogenica*, *Fusobacterium*, *Veillonella parvula*, *Porphyromonas endodontalis*, *Prevotella Pallens*, *Dialister*, *Streptococcus anginosus*, *Prevotella nigrescens*, *Campylobacter ureolyticus*, *Prevotella nanceiensis*, and *Peptostreptococcus anaerobius*	Increase within the oral cavity where oral squamous cell carcinoma is present	32783067	[130]
Oral squamous cell carcinoma	*Candida*, *Malassezia*, *Saccharomyces*, *Aspergillus*, and *Cyberlindnera*	Increase within the oral cavity where oral squamous cell carcinoma is present	34712619	[131]
Pancreatic ductal carcinoma	*Firmicutes* and *Prevotella*	Increase within the oral cavity of patients with pancreatic ductal carcinoma	35398347	[132]
	*Streptococcus salivarius*, *Streptococcus thermophilus*, and *Streptococcus australis*	Decrease within the oral cavity of patients with pancreatic ductal carcinoma		
Pancreatic cancer	*Fusobacterium periodonticum*	Increase within the oral cavity of patients with pancreatic cancer	33204698	[133]
	*Neisseria mucosa*	Decrease within the oral cavity of patients with pancreatic cancer		
Pancreatic cancer	*Porphyromonas gingivalis* and *Aggregatibacter actinomycetemcomitans*	Increase in the risk of pancreatic cancer	27742762	[134]
	Phylum *Fusobacteria* and its genus *Leptotrichia*	Decrease in the risk of pancreatic cancer		
Colorectal cancer	*Desulfovibrio desulfuricans*	Increase within the oral cavity of patients with colorectal cancer	34268367	[135]
Colorectal cancer	*Eubacterium*, *Bifidobacterium*, and *Fusobacterium*	Increase within the oral cavity of patients with colorectal cancer	36612188	[136]
Colorectal cancer	*Fusobacterium*, *Treponema*, and *Porphyromonas*	Increase within the oral cavity of patients with colorectal cancer	33052235	[137]
Esophageal squamous cell carcinoma	*Porphyromonas gingivalis*	Increase within the oral cavity of patients with esophageal squamous cell carcinoma	33201403	[138]
Esophageal squamous cell carcinoma	*Bosea*, *Solobacterium*, *Gemella*, and *Peptostreptococcus*	Increase within the oral cavity of patients with esophageal squamous cell carcinoma	34604107	[139]
Esophageal cancer	*Firmicutes*, *Negativicutes*, *Selenomonadales*, *Prevotellaceae*, *Prevotella*, and *Veillonellaceae*	Increase within the oral cavity of patients with esophageal cancer	33194789	[140]
	*Proteobacteria*, *Betaproteobacteria*, *Neisseriales*, *Neisseriaceae*, and *Neisseria*	Decrease within the oral cavity of patients with esophageal cancer		
Gastric cancer	*Campylobacter concisus*	Increase in the gastric cancer risk due to the high abundance within the tongue coating	30478535	[141]
Gastric cancer	*Streptococcus*	Increase in the gastric cancer risk due to the higher abundance within the tongue coating	30410609	[142]
	*Neisseria*, *Prevotella*, *Prevotella7*, and *Porphyromonas*	Decrease in the gastric cancer risk due to the higher abundance within the tongue coating		
Gastric cancer and colorectal cancer	*Streptococcus*, *Gemella*, *Escherichia-Shigella*, and *Fusobacterium*	Increase within the oral cavity of patients with gastric and colorectal cancers	35663463	[143]
	*Haemophilus*, *Neisseria*, *Faecalibacterium*, and *Romboutsia*	Decrease within the oral cavity of patients with gastric and colorectal cancers		
Hepatocellular carcinoma	*Streptococcus*	Increase within the oral cavity of patients with hepatocellular carcinoma	37089022	[144]
Breast cancer	*Clostridia*	Increase within the oral cavity of patients with breast cancer	37127667	[145]
Colorectal cancer	*Fusobacterium periodonticum*	Increase within the oral cavity of patients with colorectal cancer		
Lymph node metastasis	*Prevotella*, *Stomatobaculum*, *Bifidobacterium*, *Peptostreptococcaceae*, *Shuttleworthia*, and *Finegoldia*	Increase within the oral cavity of patients with lymph node metastasis in oral squamous cell carcinoma	34848792	[146]
Head and neck squamous cell cancer	*Corynebacterium* and *Kingella*	Decrease in the risk of head and neck squamous cell cancer	29327043	[147]
